# Closed-Loop Brain Devices in Offender Rehabilitation: Autonomy, Human Rights, and Accountability

**DOI:** 10.1017/S0963180121000141

**Published:** 2021-10

**Authors:** Sjors Ligthart, Tijs Kooijmans, Thomas Douglas, Gerben Meynen

**Affiliations:** 1 Department of Criminal Law, Tilburg University, Tilburg, The Netherlands; 2 Oxford Uehiro Centre for Practical Ethics, Faculty of Philosophy and Jesus College, University of Oxford, Oxford, UK; 3 Willem Pompe Institute, Utrecht University & Philosophy, VU Amsterdam, The Netherlands

**Keywords:** closed-loop brain devices, brain interventions, criminal justice, offender rehabilitation, autonomy, human rights, accountability

## Abstract

The current debate on closed-loop brain devices (CBDs) mainly focuses on their use in a medical context; possible criminal justice applications have only received incidental scholarly attention. Unlike in medicine, in criminal justice, CBDs might be offered on behalf of the State and for the purpose of protecting security, rather than realizing healthcare aims. It would be possible to deploy CBDs in the rehabilitation of convicted offenders, similarly to the much-debated possibility of employing other brain interventions in this context. Although such use of CBDs could in principle be consensual, there are significant differences between the choice faced by a criminal offender offered a CBD in the context of criminal justice, and that faced by a patient offered a CBD in an ordinary healthcare context. Employment of CBDs in criminal justice thus raises ethical and legal intricacies not raised by healthcare applications. This paper examines some of these issues under three heads: autonomy, human rights, and accountability.

## Introduction

Recently, two papers were published in this journal that address some fundamental ethical issues raised by closed-loop brain devices (CBDs) in healthcare.^1^ As Frederic Gilbert, Terence O’Brien, and Mark Cook notice, research involving CBDs—both detecting neurological patterns and delivering stimulation to avoid or diminish the effects of an unwanted neuronal event—constitute an important line of research in the field of brain–computer interfaces.^2^ Contrary to brain stimulation through open-loop devices, such as deep brain stimulation and transcranial magnetic stimulation, CBDs adapt to brain activity and provide a stimulation accordingly. A computer system registers brain activity and, if necessary, creates output that controls brain stimulation. Hence, such a CBD *registers* and *intervenes* “*autonomously*” in the subject’s brain processes.^3^ Possible fields of CBD application concern the treatment of neurological patients suffering from Parkinson disease and epilepsy. In the latter case, the device may register activity that is indicative of an upcoming seizure and, in response, may start stimulating a certain brain area in order to avoid an actual seizure.

The present debate on CBDs mainly focuses on their use in such *medical* applications; possible criminal justice applications have only received incidental scholarly attention.^4^ By contrast, the applications of other neurotechnologies that register or intervene in a person’s brain processes are widely being discussed in the context of *criminal justice.* For example, the promises and perils of registering brain activity of convicted offenders through neuroimaging technologies, such as functional magnetic resonance imaging (fMRI), for instance to yield information regarding recidivism risks,^5^ are currently under debate.^6^ Because the aim of such applications is to “read” information out of the brain, which enables drawing inferences about particular mental states, they are sometimes referred to as “*brain-reading.*” Similarly, the permissibility of deploying “*brain interventions*” in convicted offenders, such as pharmaceuticals and (non)invasive brain stimulation, aiming to enhance particular brain processes and preventing reoffending, has been subjected to an extensive ethical debate.^7^ Apart from being discussed in the literature, both brain-reading and brain interventions have actually been employed in criminal justice settings, for example to obtain brain data in the context of the insanity defence (fMRI),^8^ or to prevent recidivism among sex offenders (chemical castration).^9^ Whereas both debates on registering and intervening with brain processes in the context of criminal justice are usually conducted separately, the development of CBDs makes it important to consider registration and intervention *together.* This is especially the case as the combination of these technologies in a closed-loop brain device entails further ethical, but also legal intricacies, for example, concerning the user’s autonomy.

At least in theory, it would be possible to employ CBDs in criminal justice, and, more specifically, in the rehabilitation of convicted offenders, similarly to the much-debated possibility of using brain interventions in this context. As Frederic Gilbert notes with regard to “predictive brain implants,” they

could in theory be utilized for treating a limited number of other brain disturbances where evidence shows that neuronal changes happen prior to symptoms occurring. As an illustration of how brain activity could be detected and stopped before manifesting itself, we can think of certain forms of aggressive and violent behaviors working in a similar manner to the onset of epilepsy, in particular temporal or frontal lobe seizures, as well impulsive sexual urges.^10^

Arguably, aggressive and sexual impulses are the most important ones to diminish from a criminal law point of view. In general, CBDs are likely to be most helpful for disturbances that are not always present, but come and go. This is, generally, true for impulse control disorders, which can, but need not be related to aggression and sex. Other types of impulse-control related behaviors that are relevant for criminal law are pyromania and kleptomania.

Unlike in medicine, in criminal justice, CBDs might be offered on behalf of the State for safety reasons, instead of the aim of healthcare. Notably, although such use of CBDs is in principle consensual—the offender has a choice to either accept or decline the offer—the context of criminal justice and the type of choice the offender is confronted with (for instance: parole in exchange for CBD use) are different from CBD use in healthcare. Obviously, such employment of CBDs in criminal justice raises additional fundamental ethical and legal questions—even when CBDs might be noninvasive. In this paper, we consecutively address the following three issues: autonomy, human rights, and accountability. The legal context for our analysis of human rights is provided by the European Convention on Human Rights (ECHR). Throughout, we assume that one purpose of CBDs used in criminal justice settings would be to prevent an offender from recidivating (“correctional CBDs”), although we leave it open whether this goal would be sought for the benefit of the offender, the benefit of others, or both.

## Autonomy

As noted in the introduction, the current debate on CBDs largely focuses on the use of these technologies in a medical context. However, the discussion on healthcare CBDs and autonomy is also relevant to the context of *offender* rehabilitation.

As to autonomy, the present debate distinguishes between healthcare CBDs that operate completely independent of the patient’s will, entirely taking the patient *out of the decisional loop*, and CBDs that provide patients with feedback and give them some control over the system, keeping them *in the decisional loop.* ^11^ Whereas the out-of-the-loop-patient has no control over decisions on whether and how to react in response to particular neuronal events—“outsourcing” those decisionmaking tasks to the CBD’s algorithms—it has well been argued that the in-the-loop-subject retains significant autonomy over decisionmaking.^12^ Moreover, some have suggested that CBDs that keep the patient in the decisional loop, providing assistive guidance in choosing how to act and (thus) enlarging one’s range of choice, can be interpreted by the patient as an integral component of his increased degree of control, which leads to his (increased) sense of autonomy.^13^ At the same time, over-relying on the information provided by the CBD might *de fa*cto also result in outsourcing the patient’s decisionmaking, adversely affecting one’s autonomy.^14^ Whether outsourcing decisionmaking tasks does indeed necessarily diminish autonomy, is open for debate.^15^

The impact on autonomy, we argue, will also depend on our understanding of the concept. If autonomy is conceptualized as the absence of certain forms of interference or of control by others or by algorithms,^16^ any CBD in criminal justice, employed on behalf of the State would probably diminish autonomy. The reason is that the device is able to interfere with or to circumvent a person’s own decisionmaking.

But there are other ways of interpreting autonomy. For instance, if autonomy consists in having control over one’s own life, then whether CBD—either consensual or nonconsensual—decreases autonomy will depend on whether the mental states suppressed by the device are themselves states that interfere with the person’s control over one’s life. For example, suppose the rehabilitation of an offender has failed several times because of behavioral patterns that he apparently cannot shake off—much to the offender’s own regret. In this way, he will never be able to build the life in the community he desires. If a correctional CBD targets those mental/brain states that undermine the offender’s rehabilitation, the CBD might be considered to empower the offender, increasing his autonomy, at least in the sense of having control over one’s own life. Whereas the first view of autonomy focuses on the relationship between other agents and one’s individual decisions, the second view looks at the autonomous life from the inside, focusing on an individual’s internal capacities and powers.

Such a positive effect on autonomy—in the sense of controlling one’s own life—might be stronger for the in-the-loop-subject compared to the out-the-loop-subject. Apart from contributing to supressing autonomy-undermining mental states, in-the-loop-CBDs could provide the individual with options he would not otherwise had had, such as the opportunity to extinguish an impending urge.^17^ One could imagine a process where the offender gets more and more in the loop during the process. The more robust and successful the rehabilitative steps, the more an offender can take matters in his own hand. Gradually, the offender enters the loop. Of note, he is only out of the loop regarding a very limited type of behavioral choices, namely the choices that potentially—yet profoundly—interfere with his aim: successful re-entering society.

In discussing the issue of autonomy, the question arises whether the impact of correctional CBDs would be different compared with other brain interventions, such as pharmaceuticals and deep brain stimulation. Take the case of an offender suffering from a sexual disorder, who has been convicted for sexual assault. Would the impact on the offender’s autonomy be different if the judge imposes correctional CBD compared with chemical castration, both targeting the offender’s sexual arousal? If autonomy consists in controlling one’s own life, the answer to this question would probably depend on whether the offender is in, or out of the decisional loop. If the latter, the CBD controls the offender’s mental states similar to chemical castration: both targeting sexual arousal while completely bypassing the offender’s will. By contrast, if the CBD keeps the offender in the decisional loop, the offender keeps at least some ultimate control over whether and how to react in response to the identified neuronal event.

If autonomy is equated (in part) with the absence of certain forms of interference or control by others, correctional CBDs will, in general, probably have a limited adverse effect on autonomy compared with other brain interventions. This is because CBDs are smart in the sense that they interfere with the subject’s mental states *only* at particular times, when it is necessary to avoid or diminish the effects of an unwanted neuronal event. For example, in the case of the sexual offender, a CBD may only provide stimulation if it identifies (brain correlates of) sexual arousal. By contrast, chemical castration delimits sexual arousal 24/7, constantly controlling the offender’s hormones and exposing him to various side-effects. Similarly, deep brain stimulation constantly stimulates the targeted brain area. Hence, compared to such more traditional brain interventions, CBDs ability to interfere with the offender’s mental states is more subtle and focused; only when controlling a specific neuronal event is necessary. In our view, this should be a relevant consideration in discussing the possibilities and perils of CBDs and other brain interventions for the rehabilitation of criminal offenders.

## Human Rights

In view of the advances in neurotechnology and the possibilities it offers both for the registering of, and intervening with particular brain processes, some scholars have argued to explicitly recognize novel legal rights over the mind, such as rights to mental integrity, cognitive liberty, mental self-determination, and mental privacy.^18^ At the same time, others have argued that, at least in the European context, legal protection against brain-reading and brain intervention is already enshrined in the current framework of human rights, most prominently under the right to freedom of thought and the right to respect for private life.^19^ In what follows, we briefly discuss these existing legal rights, and explore their possible implications for CBDs employed in order to prevent an offender from recidivating (correctional CBDs).

### Freedom of Thought

According to Article 9 ECHR, everyone has the right to freedom of thought, conscience, and religion, including the freedom to change and manifest one’s religion or belief, for example, through engaging in religious practices. This right comprises an internal and external dimension. Whereas the freedom of thought, conscience and religion concerns the internal dimension, the right to manifest one’s religion and belief comprises the external dimension.^20^

Although the external dimension is relative (it can be restricted in specific circumstances), the internal dimension of freedom of thought is absolute, in that it may never be lawfully infringed. The absolute internal dimension guarantees that the State may never interfere with the most intimate and inner sphere of its citizens, neither by imposing coercive measures to make a person *change* what he believes, nor by using inquisitorial methods to *discover* one’s personal thoughts and convictions.^21^ As the “founding fathers” of Article 9 ECHR put it, the right to freedom of thought, conscience, and religion intends to protect “not only from ‘confessions’ imposed for reasons of State, but also from those abominable methods of police enquiry or judicial process which rob the suspect or accused person of control of his intellectual faculties and of his conscience.”^22^

At first glance, freedom of thought, protecting from registering and controlling of thoughts, seems to offer clear legal protection with regard to CBDs—both registering and controlling particular brain processes of the individual. However, the Grand Chamber of the European Court of Human Rights (the Court) held that “the right to freedom of thought, conscience and religion denotes only those views that attain a certain level of cogency, seriousness, cohesion, and importance.^23^ Therefore, it is not clear at all that the data CBDs register, and the brain processes they (aim to) control, constitute “thoughts” in the meaning of Article 9 ECHR. Presumably, this will depend on the precise details of the application. For example, do emotions qualify as “thoughts” narrowly defined under Article 9 ECHR?^24^ As Evans notes, the right to freedom of thought and conscience basically comprises personal thoughts on political, philosophical, ethical, and intellectual positions in human affairs.^25^ Whereas correctional CBDs may well register and control “important” mental states, like an upcoming epileptic seizure or (potentially) sexual arousal, it is less clear whether such mental states should be considered as attaining “a certain level of cogency, seriousness and cohesion,” tantamount to political preferences and philosophical positions. Hence, whether correctional CBD falls within the scope of the absolute right to freedom of thought, is yet an open question.

In order to guarantee fundamental legal protection from novel technologies that register and intervene in brain processes, some have argued that the scope of freedom of thought ought to be broadened so as to cover *any mental state* that has “content or meaning.”^26^ At the same time, as Christoph Bublitz argues, in some cases good reasons can exist to control or disclose particular mental states of an individual. For example, (nonconsensual) psychiatric interventions in order to change the patient’s mental states are sometimes necessary to protect the wellbeing of the patient, e.g. in the context of suicidal depression. And sometimes, individuals have legal duties to speak and disclose particular mental states, such as a witness in court.^27^ Therefore, some authors suggest that there is a need to reconsider the absolute nature of freedom of thought, and develop (narrow) exceptions that might justify the registering and controlling of mental states.^28^

### Freedom of Expression

However, whereas reconsidering freedom of thought may ensure fundamental legal protection regarding correctional CBDs, it could well be the case that, at present, other fundamental rights already offer legal protection in this respect. For example, Article 10 ECHR guarantees freedom of expression, including the “freedom to hold opinions and to receive and impart information and ideas without interference by public authority.” Interestingly, Article 10 ECHR has been understood to include a right *not* to convey opinions, ideas and other information.^29^ Whereas freedom of *thought* only protects particular mental states, attaining a certain level of cogency, seriousness, cohesion, and importance, the scope of freedom of (non)*expression* is considered “extremely broad,”^30^ encompassing not only the substance of (almost any) information and idea, but also protecting “a diverse variety of forms and means in which they are manifested, transmitted, and received.”^31^

Still, whether registering and controlling the manifestation of, for example, sexual arousal with CBDs would fall within the scope of this right remains unsure, because it is not clear that the signals sent to the CBD would count as “expression,” nor whether the CBD’s intervention affects “opinions” or “ideas” in the meaning of Article 10 ECHR. Meanwhile, prior to reconsidering fundamental rights such as the right to freedom of thought, an in-depth examination of the legal implications of (among other things) correctional CBDs in light of existing fundamental rights is desirable.

Whereas it is yet to be examined whether the employment of correctional CBDs falls within the scope of the right to freedom of thought and of (non)expression, it is clear that the generic right to respect for private life offers (at least some) protection is this respect, which is discussed below.

### The Right to Respect for Private Life

Under Article 8(1) ECHR, everyone has the right to respect for his private life. According to the Court, “private life” is a broad concept that does not lend itself to an exhaustive definition.^32^ Therefore, and since the Court approaches the ECHR as a living instrument that should be interpreted in light of present-day conditions,^33^ the scope of the right to respect for private life is constantly evolving, for example in the light of technological and ethical developments. Notwithstanding difficulties defining “private life,” it is clear that it covers the individual’s (1) personal data, (2) bodily integrity, and (3) psychological and moral integrity.^34^

Since CBDs register information from the brain of the individual, for example regarding a psychiatric disorder or sexual arousal, such devices register information “relating to an identified or identifiable individual”—that is, they process (sensitive) personal data. The collection, storage, and disclosure of such data without consent infringes the right to respect for private life, as will its use, for example, in a legal procedure.^35^ Whether the processing of personal data through correctional CBDs qualifies as “collecting, storing and/or disclosing” personal information, will probably depend on whether the data will become available for a third party (e.g., the probation officer)—that is, whether the information is “extracted” from the CBD user. If the CBD only moves brain data from one “part of the brain” to another, it seems not to interfere with any considerable personal data interest of its user.

Clearly, if a CBD is placed within the brain of the subject through brain surgery, it infringes the individual’s bodily integrity. But even if CBDs could be employed without such bodily intrusion — registering and controlling brain processes from outside the skull — the fact that it aims to intervene in and/or change physical reactions in the brain, would presumably constitute an interference with the individual’s bodily integrity anyway, infringing the right to respect for private life.^36^

As to physiological and moral integrity, the precise meaning and scope of these notions are unclear. Although the Court indicates that both “moral” and “psychological” integrity are interchangeable terms, it refrains from providing a precise definition.^37^ What is clear though, is that a person’s mental health is a crucial part of private life associated with the aspect of moral integrity.^38^ Furthermore, together with bodily integrity, the individual’s psychological integrity comprises “multiple aspects of the person’s identity such as, for example, gender identification, sexual orientation, name, and elements relating to a person’s right to his or her image.”^39^ Employing CBDs to “enhance” the brains of convicted offenders by intervening with particular mental states has the potential to affect the subject’s mental health. Therefore, it it seems plausible to assume that the Court would be inclined to consider such correctional CBDs as interfering with moral integrity, thus (again) infringing the right to private life. In addition, one could raise the question of whether imposing a CBD upon an individual, influencing her brain processes, interferes with the individual’s (narrative) identity.^40^

Note, however, that *infringements* of the right to private life do not necessarily imply a *violation* of this right. An infringement with the rights guaranteed by Article 8(1) ECHR *can* be justified if it complies with the requirements of Article 8(2) ECHR—that is, if it is in accordance with the law and necessary in a democratic society for the legitimate interest of, *inter alia*, the prevention of crime. Although this justification clause is often considered to be broad, potentially justifying a wide range of infringements, it is not clear at all that correctional CBDs would be permissible. For example, in assessing whether an infringement with Article 8 ECHR is necessary for the prevention of crime, the Courttakes into account whether the case raises sensitive moral or ethical issues: if that is the case, the level of discretion (“margin of appreciation”) afforded to the Member States to balance the competing interests at stake, tends to be broader.^41^ Hence, if CBDs enter the domain of criminal justice, the law, ideally informed by ethics, should develop a normative approach under Article 8 ECHR, tailored to the particularities of CBDs.

## Accountability

When human subjects and (semi)autonomous systems intertwine with each other, we must consider how to approach moral and legal accountability.^42^ As to brain interventions, such as pharmaceuticals and deep brain stimulation, the issue of accountability has gained considerable scholarly attention.^43^ In this regard, the main question in the moral-philosophical and legal literature focuses on whether and, if so, to which extent an individual can be held morally and legally accountable for actions that follow from the modification of one’s brain. For example, whereas *P* used to be a law-abiding citizen before his brain-intervention, thereafter and as a result thereof, *P* has turned into a violent, strongly pleasure-seeking person. Acting from these traits, *P* rapes *X.* To what extent can *P* be held morally and legally accountable for his actions?

In the present paper, we do not discuss this particular question (stemming from the healthcare context). Instead, we explore how the employment of correctional CBDs sheds new light on the present debate on accountability for actions that (partially) result from brain intervention. We focus on the following question, as was raised by Philipp Kellmeyer et al. regarding CBD-treatment of epilepsy:

What if a medical device fails to predict and interrupt an epileptic seizure, which results in the subject being in an unsafe environment, leading to her injury and/or the injuries of others? Who will be taken to court—the subject, the programmer, or the device company?^44^

Imagine the case of *P*, who is convicted for sexual child abuse. In the context of his conviction, the judge offered a CBD, registering *P*’s sexual arousal and, if necessary, automatically intervening to suppress it. The CBD initially works well, however, after 4 years of being a “perfect” citizen, *P* reoffends: while he is waiting in the queue to pay for his groceries, a system error occurs. The CBD fails to register *P*’s sexual arousal and (thus) does not intervene, from which arousal *P* subsequently touches a child.

To what extent is *P* criminally accountable for his action? The answer to this question may well vary depending on whether the approach is legal or moral-philosophical.^45^ Yet, in what follows, we discuss three issues that we believe would be relevant under both a legal and moral-philosophical approach—that is, whether (I) the system failure could be qualified as a “mental impairment,” (II) the CBD operates through keeping the subject in or out the loop, and (III) the CBD is self-learning.

### Mental Impairment

Both in criminal law and philosophy, it is arguably accepted that under certain circumstances, a mental impairment at the time of the offence can diminish responsibility or (completely) excuse an offender for his actions through an insanity defence.^46^ Although the precise requirements vary across different legal systems, many jurisdictions recognize some form of the insanity defence.^47^ For example, in England, according to the M’Nagthen Rules a defendant will not be held criminally responsible if, “at the time of the committing of the act, he was laboring under such a defect of reason, from disease of the mind, as not to know the nature and quality of the act he was doing; or if he did know it, that he did not know he was doing what was wrong.” Meanwhile, in the United States, according to the Model Penal Code Test, one “is not responsible for criminal conduct if at the time of such conduct as a result of mental disease or defect he lacks substantial capacity either to appreciate the criminality (wrongfulness) of his conduct or to conform his conduct to the requirements of the law.”

As to the case of *P*, whose criminal offence resulted from a defect in his CBD, the question arises whether such a system error could, or should be qualified as a disease of the mind (“mental impairment”) in the meaning of the insanity defence. If so, *P* could potentially be (partly) excused for his actions—depending on the precise requirements of the insanity defence at issue.

A simple argument in favor of exculpation or diminished responsibility could appeal to the fact that the malfunction of the CBD diminishes *P*’s ability to control his sexual arousal, and thus to conform his conduct to the requirements of the law, compared to the prior situation where the CBD was functioning normally.

However, to counter this argument, one could argue that, during the time that the CBD was functioning normally, *P* enjoyed *enhanced* capacity to control his arousal. Thus, it may be that the malfunction merely takes him back to his pre-CBD level of capacity, which may have been sufficient for full accountability. If *P*’s control over his conduct was impaired before employing the CBD, a subsequent system failure will bring *P* back in his original state of mental impairment. In that case, it is not the error itself that should qualify as mental impairment, but rather *P*’s own authentic (impaired) mental state. In such cases, exculpation through the insanity defence will still be possible if, as a result of the impaired original mental state, the subject, for example, lacked the capacity to appreciate the wrongfulness of his conduct or to conform his conduct to the requirements of the law. In cases like that, the system failure itself is not significant. Instead, a causal relationship should be determined between the individual’s action on the one hand and, on the other hand, his (own) mental impairment.

However, one could argue that if the CBD user reasonably relies on the CBD, and then all of a sudden it stops working, the user effectively has a new impairment that he did not have to begin with: an inability to know in advance how the CBD, and thus his own mind, is going to behave. This would make it more difficult for the individual to control his conduct compared with a situation he had no CBD at all: not only is his control reduced by the loss of the control-enhancing effect of the CBD, it is also reduced by his inability to predict his own mental responses.

But what if the system failure does not merely result in the CBD not working, but the device malfunctions in a way resulting in erratic brain stimulation, from which stimulation the subject causes harm to another? The situation would arguably be similar to one in which a person suddenly starts to suffer from epilepsy, as a result of which a part of his brain becomes overactive and causes involuntary movements from time to time. In such a case, the system failure itself *does* cause a disturbance of the subject’s mental state. Hence, it would be less problematic to argue that errors like these should qualify as mental impairment that diminishes or negates the subject’s accountability.

As the above discussion illustrates, determining whether there is a mental impairment of a sort that might undermine accountability will be a complex matter in cases of a malfunctioning CBD. It will depend, among other things, on (1) the individual’s level of capacity before receiving the CBD, (2) the degree to which the CBD enhances capacity, and (3) whether the malfunction of the CBD introduces a new incapacity or merely removes the enhancement of capacity that it provided.

### In or Out the Loop

As discussed in the “Autonomy” section, the in-the-loop-subject may have increased autonomy compared to the out-of-the-loop-subject. As a consequence, Philipp Kellmeyer et al. note, the in-the-loop-subject has increased accountability as well.^48^ They argue:

The out-of-the-loop subject has some responsibility for consenting to the consequences of the implanted system but is not accountable for the consequences of any particular seizure. If, on the other hand, the subject remains in the loop (e.g., via a visual feedback system), the subject’s failure to modify her behavior in accordance with the indicated level of risk may indeed result in moral and legal accountability.

First, concerning the out-of-the-loop-subject, in the context of criminal justice, where a CBD might be *imposed* upon the convicted offender, accountability for *consenting* to the potential consequences of the CBD is not relevant.^49^ This could be different if a CBD is not imposed on behalf of the State, but offered to the offender as an alternative to, for example, prison, or without formal strings attached. However, even then, questions could be raised regarding whether an offender is accountable for any consequences of the CBD, since the offender may have agreed to undergo it only out of desperation to avoid further prison time. (Even if there is no formal link made between agreement to undergo the CBD and early release, the offender may know that agreeing to receive the intervention is likely to lower the risk he is deemed to pose and may therefore be helpful in securing release, for example, via parole.)

Second, in light of the in-the-loop-subject’s increased autonomy,^50^ any failure of the *subject* to modify his behavior in accordance with the communicated output of the CBD, resulting in harm to others, might indeed not justify exculpation. After all, within his increased autonomy, the subject should be held accountable for his decision to ignore the communicated output. However, what if it is not the subject, but the CBD that fails, by not registering the relevant brain process and, subsequently, not providing (the right) output to the subject? Could the in-the-loop-subject’s autonomy still be considered to be increased in such cases and, if so, should this lead to (or increase) his accountability for not acting differently? In our view, if the user might reasonably rely on the CBD, it would not be unreasonable to argue that any *unforeseeable* technological failure resulting in a criminal offence should reduce accountability.

In addition, if the CBD fails to provide the in-the-loop-subject with information to act upon, the question arises whether that subject should still be treated differently in terms of accountability compared to the out-of-the-loop-subject. After all, both subjects are not in the loop (anymore), either because one has never been in the loop, or because the loop is discontinued by the occurred error.

### Self-Learning Technology

Finally, whether and, if so, who should be accountable for actions that follow from a CBD system failure may also depend on whether the CBD is self-learning. This is especially relevant (also in a broader context) in this artificial-intelligence-era where many machines are artificial intelligence (AI) driven and have self-learning capacities. The following example—with regard to robots—may help to illustrate this.

Suppose that a robot wrongfully identifies a human being (*X*) as a threat to his objective. Somehow, it “thinks” *X* is in its way. Because this robot is smart, self-learning, and self-deciding, it calculates the most efficient way to eliminate the threat. So the robot decides to use its hydraulic arm to push *X* into an adjacent machine. This machine is heavy industrial machinery, thus killing *X* instantly. This prompts the question: who is to blame? We are not dealing with a merely hypothetical situation here. It actually happened in Japan and this is only one of many examples that show the dangers of AI.^51^

The ability of an AI-entity to act is brought about by its capability to gain experiences and to learn from them.^52^ This feature makes AI-entities interesting for many authorities. But the example of the robot illustrates the perils of employing self-learning technology in daily life. CBDs both detect neurological patterns and deliver stimulation to avoid or diminish the effects of an unwanted neuronal event by adapting to brain activity and providing a stimulation accordingly. If CBDs are self-learning, the issue of foreseeability becomes even more complicated. The specific way in which such CBDs might adapt, and learn to better adapt to brain activity will by definition not be (completely) foreseeable, not even to the institution that created the device. In this regard, it should be borne in mind that the Member States of the ECHR have the positive obligation to take measures designed to ensure that individuals within their jurisdiction are not subjected to torture or inhuman or degrading treatment, including such ill-treatment administered by private individuals.^53^ Therefore, the judiciary can only employ CBDs if they have no reason to distrust the reliability of these devices. This calls for a critical assessment of their reliability, the more so if CBDs are self-learning.

Harmful actions (defined as criminal offences in a Criminal Code) that are “committed” by AI-entities, which have been triggered by (the failure) of self-learning devices, may need to be imputed to a legal and/or natural person. In order to impute the action of person *A* to person *B*, it should be established that *B* had the power to control whether this action (or similar actions) would actually take place, and that *B* accepted this action (or similar actions).^54^ This twofold criterion requires foreseeability of *A*’s actions in order to establish criminal liability with regard to *B.* As such, the criterion functions adequately in the “classical” legal domain in which actions of (natural or legal) person *A* are imputed to (natural or legal) person *B.* However, it is uncertain whether *foreseeability* and *control* can be established in case of self-learning and self-deciding machines.

In addition, in order to be punishable a criminal offence must be committed intentionally or be the consequence of negligence. If the CEO (*C*) of a company is aware of illegal behavior of his employee *D*, but he does not take action to stop this behavior, *C* can be held criminally liable for the offence, intentionally committed by *D.* In case of AI-driven technology, it is not certain—to say the least—whether a natural or legal person can be said to have acted intentionally or with negligence if the device (as a result of its self-learning abilities) acted in a way that could not reasonably have been foreseen.

In short, if a (self-learning) CBD intervenes or omits to intervene in a way that has harmful consequences, it is not evident that the carrier of the CBD can be “automatically” held criminally accountable for those consequences, especially in case of an out-of-the-loop-CBD. The same can be said with regard to the manufacturer of the CBD and the company providing for its software. The problem of the imputation of actions with harmful consequences to (other) persons therefore needs to be thought through in a very thorough manner.

## Conclusion

Whereas the current debate on CBDs focuses on consensual employment in healthcare, we explored some ethical and legal issues regarding *correctional CBDs.* Similar to the much-debated brain-reading and brain altering techniques, correctional CBDs might potentially contribute to preventing crime, or more generally facilitating the rehabilitation of convicted offenders. Yet, such use of CBDs—even if this use would take place on a consensual basis—entails further ethical and legal intricacies, both compared with healthcare CBDs and neurotechnologies that *either* “read” *or* intervene in the brain. Anticipating technological developments, we argue that extrapolating the debate on CBDs to the context of criminal justice would be highly desirable. Not least because employing neurotechnologies in criminal justice is no “neuroscience fiction” anymore, and correctional CBDs might, in certain ways, be less intrusive than “traditional” interventions, such as chemical castration and constant brain stimulation. In this paper, we identified some ethical and legal considerations that may be helpful in pursuing the debate on CBDs tailored to the context of criminal justice.

## Notes

1.

Gilbert F , O’Brien T , Cook M . The effects of closed-loop brain implants on the autonomy and deliberation. Cambridge Quarterly of Healthcare Ethics 2018;27:316–252950912810.1017/S0963180117000640; Kellmeyer P , Cohcrane T , Müller O , Mitchell C , Ball T , Fins JJ , et al. Effects of closed-loop medical devices on the autonomy and accountability of persons and systems. Cambridge Quarterly of Healthcare Ethics 2016;25:623–332763471410.1017/S0963180116000359.

2. See note 1, Gilbert et al. 2018, at 316.

3. See note 1, Gilbert et al. 2018, at 316; Kellmeyer et al. 2016, at 623.

4. Gilbert F, Dodds S. Is There Anything Wrong With Using AI Implantable Brain Devices to Prevent Convicted Offenders from Reoffending. In Vincent NA, Nadelhoffer T, McCay A, eds. Neurointerventions and the Law: Regulating Human Mental Capacity. New York: Oxford University Press; 2020.

5.

Aharoni E , Vincent GM , Harenski CL , Calhoun VD , Sinnott-Armstrong W , Gazzaniga MS , et al. Neuroprediction of future rearrest. PNAS 2013;110(15);6223, at 282353630310.1073/pnas.1219302110PMC3625297.

6.

Meynen G. Brain-based mind reading in forensic psychiatry: Exploring possibilities and perils. Journal of Law and the Biosciences 2017;4(2);311–29; Ligthart S. Coercive neuroimaging, criminal law and privacy: A European perspective. Journal of Law and the Biosciences 2019;6(1):289–309 3166697010.1093/jlb/lsz015PMC6813934.

7.

Douglas T. Moral enhancement. Journal of Applied Philosophy 2014;25(3):228–4810.1111/j.1468-5930.2008.00412.xPMC261468019132138; Birks D , Douglas T , eds. Treatment for Crime: Philosophical Essays on Neurointerventions in Criminal Justice. New York: OUP; 2018 .

8.

Catley P , Claydon L. The use of neuroscientific evidence in the courtroom by those accused of criminal offenses in England and Wales. Journal of Law and the Biosciences 2015;2(3):510–492777421110.1093/jlb/lsv025PMC5034405; De Kogel CH , Westgeest EJMC. Neuroscientific and behavioral genetic information in criminal cases in the Netherlands. Journal of Law and the Biosciences 2015;2(3):580–605 2777421310.1093/jlb/lsv024PMC5034396.

9.

Douglas T, Bonte P, Focquaert F, Devolder K, Sterckx S. Coercion, incarceration, and chemical castration: An argument from autonomy. Bioethical Inquiry 2013;10:393–405 2381332410.1007/s11673-013-9465-4PMC3824348.

10.

Gilbert F . A threat to autonomy? The intrusion of predictive brain implants. Ajob Neuroscience 2015;6(4):9 10.1080/21507740.2015.1076087PMC468560426740906.

11. See note 1, Gilbert et al. 2018; Kellmeyer et al. 2016.

12. See note 1, Gilbert et al. 2018; Kellmeyer et al 2016; See note 9, Gilbert 2015; Meynen G , Widdershoven G . The impact of closed-loop DBS on agency: An open question. Ajob Neuroscience 2017;8(2):79–80 .

13. See note 1, and 9, Gilbert et al. 2018, at 321; Gilbert 2015, at 7–9.

14. See note 1, Gilbert et al. 2018, at 322.

15.

Douglas T . Enhancement and desert. Politics, Philosophy & Economics 2019;18(1):13–710.1177/1470594X18810439PMC638045930886539.

16.

Oshana M . How much should we value autonomy? Social Philosophy and Policy 2003;20(2):99–126 .

17. See note 10, Gilbert 2015, at 9; See note 1, Kellmeyer et al. 2016, at 627. Cf. Burwell S , Sample M , Racine E . Ethical aspects of brain computer interfaces: A scoping review. BMC Medical Ethics 2017;18(1):6 2912194210.1186/s12910-017-0220-yPMC5680604.

18.

Ienca M , Andorno R . Towards new human rights in the age of neuroscience and neurotechnology. Life Sciences, Society and Policy 2017;13(5):1–27 10.1186/s40504-017-0050-1PMC544756128444626; Lavazza A . Freedom of thought and mental integrity: The moral requirements for any neural prosthesis. Frontiers in Neuroscience 2018;12:82 2951535510.3389/fnins.2018.00082PMC5825892.

19.

Bublitz JC . Freedom of thought in the age of neuroscience, Archiv Für Rechts- Und Sozialphilosophie 2014;100:1–25 ; See note 6, Ligthart 2019.

20. Note that Article 9 ECHR does not cover the manifestation of thoughts. Yet, thoughts can be “manifested” through actions, for example, via speech and expression, covered by the freedom of expression pursuant to Article 10 ECHR.

21. Vermeulen B, Roosmalen M. Freedom of thought, conscience and religion. In: Van Dijk P et al. eds., *Theory and Practice of the European Convention on Human Rights.* Cambridge: Intersentia; 2018, at 738.

22. European Commission of Human Rights, *Preparatory work on Article 9 of the European Convention on human rights.* Strasbourg: European Commission of Human Rights; 1959, at 3–4.

23. ECtHR 1 July 2014, appl.no. 43835/11 (*S.A.S./France*), § 55.

24. See note 18, Bublitz 2014, at 18.

25. Evans C. *Freedom of Religion Under the European Convention on Human Rights.* New York: OUP; 2001, at 52.

26. See note 19, Bublitz 2014, at 18; McCarthy-Jones S. The autonomous mind: The right to freedom of thought in the twenty-first century. *Frontiers in Artificial Intelligence* 2019;2:19.

27. See note 19, Bublitz 2014, at 10, 19–20, 24–5.

28. See note 19 and 26, Bublitz 2014 and McCarthy-Jones 2019. See also Ligthart, S. Freedom of thought in Europe: Do advances in brain-reading technology call for revision? *Journal of Law and the Biosciences* 2020, lsaa048.

29. ECtHR 23 October 2018, appl.no. 26892/12 (*Wanner/Germany*); ECtHR 3 April 2012, appl.no. 41723/06 (*Gillberg/Sweden*), § 86.

30.

Rainey B , Wicks E , Ovey C . The European Convention on Human Rights. New York: OUP; 2017, at 483 .

31.

Harris DJ. Harris, O’Boyle, and Warbrick: Law of the European Convention on Human Rights. New York: OUP; 2018, at 594 .

32. ECtHR 25 September 2018, 76639/11 (*Denisov/Ukraine*), § 95.

33. ECtHR 19 February 2013, appl.no. 19010/07 (*X and others/Austria*), § 139.

34. For example, ECtHR 8 November 2016, appl.no. 18030/11 (*Magyar Helsinki Bizottság/Hungary*), § 191; ECtHR 25 September 2018, appl.no. 76639/11 (*Denisov/Ukraine*), § 95; ECtHR 24 July 2014, appl.no. 7446/12 (*Remetin/Croatia (No. 2)*), § 70.

35. See note 31, Harris et al. 2018, at 538.

36. Cf. EComHR 10 December 1984, appl.no. 10435/83 (*Acmanne and others/Belgium*).

37.

De Vries K. Right to respect for private and family life. In Van Dijk P et al. eds., Theory and Practice of the European Convention on Human Rights. Cambridge: Intersentia; 2018, at 690 .

38. ECtHR 26 November 2009, appl.no. 25282/06 (*Dolenec /Croatia*), § 165.

39. ECtHR 29 March 2016, appl.no. 56925/08 (*Bédat/Switzerland*), § 72.

40.

Aggarwal S , Chugh N. Ethical implications of closed loop brain device: 10-year review. Minds & Machines 2020;30:145–70, at 159.

41. ECtHR 24 January 2017, appl.no. 25358/12 (*Paradiso and Campanelli/Italy*), § 182.

42. See note 1, Kellmeyer et al. 2016, at 629.

43. For example, De Marco G. Brain interventions, moral responsibility, and control over one’s mental life. *Neuroethics* 2019;12:221–9; Bublitz C, Merkel R. Guilty minds in washed brains? Manipulation cases and the limits of neuroscientific excuses in liberal legal orders. In: Vincent NA ed., *Neuroscience and Legal Responsibility.* New York: OUP; 2013.

44. See note 1, Kellmeyer et al. 2016, at 629.

45. See note 43, Bublitz, Merkel 2013, at 333–4.

46.

Sinnott-Armstrong W , Levy K. Insanity defenses. In Deigh J , Dolinko D , eds., The Oxford Handbook of Philosophy of Criminal Law. New York: OUP; 2011 .

47.

Simon RJ , Ahn-Redding H. The Insanity Defense, the World Over. Lanham: Rowman & Littlefield Publishers; 2006 .

48. See note 1, Kellmeyer et al. 2016, at 630.

49. In addition, it is debatable whether any system failure will be foreseeable, and (thus) whether the subject can be considered to have consented to such a failure.

50. Because the CBD then provides the subject as much information as possible about one’s brain processes, enhancing his ability to control one’s own behavior.

51.

Weng Y , Chen C , Sun C. Towards the human-robot co-existence society: On safety intelligence for next generation robots. International Journal of Social Robotics 2009;1:267–82.

52.

Bundy A , Ohlsson S. The nature of AI principles. In: Patridge D , Wilks Y , eds., The Foundation of Artificial Intelligence. New York: Cambridge University Press; 2006, at 135 .

53.

Akandji-Kombe J. Positive obligations under the European Convention on Human Rights. Strasbourg: Council of Europe; 2007, at 28 .

54. See, for example (for the Dutch legal context): De Hullu J. Materieel strafrecht. Deventer: Wolters Kluwer; 2018, at 163 .

